# Monitoring Response and Resistance to Treatment in Chronic Lymphocytic Leukemia

**DOI:** 10.3390/cancers16112049

**Published:** 2024-05-28

**Authors:** Ilaria Del Giudice, Irene Della Starza, Filomena De Falco, Gianluca Gaidano, Paolo Sportoletti

**Affiliations:** 1 Hematology, Department of Translational and Precision Medicine, Sapienza University, 00161 Rome, Italy; dellastarza@bce.uniroma1.it; 2 AIL Roma, ODV, 00161 Rome, Italy; 3 Department of Medicine and Surgery, Institute of Hematology and Center for Hemato-Oncological Research, University of Perugia, 06129 Perugia, Italy; filomena.defalco@unipg.it; 4 Division of Hematology, Department of Translational Medicine, Università del Piemonte Orientale, 28100 Novara, Italy; gianluca.gaidano@med.uniupo.it

**Keywords:** chronic lymphocytic leukemia, BTK inhibitors, BCL2 inhibitors, response criteria, measurable residual disease, resistance

## Abstract

**Simple Summary:**

Novel targeted agents in chronic lymphocytic leukemia (CLL) are employed with different strategies. The evolution of response assessment criteria of the clinical application of measurable residual disease (MRD) analysis and the novel genetic and non-genetic mechanisms of resistance according to the novel treatment strategies within clinical trials are reviewed. Our view on how this knowledge can be applied to the current and future real-life management of CLL patients is discussed.

**Abstract:**

The recent evolution in chronic lymphocytic leukemia (CLL) targeted therapies led to a progressive change in the way clinicians manage the goals of treatment and evaluate the response to treatment in respect to the paradigm of the chemoimmunotherapy era. Continuous therapies with BTK inhibitors achieve prolonged and sustained control of the disease. On the other hand, venetoclax and anti-CD20 monoclonal antibodies or, more recently, ibrutinib plus venetoclax combinations, given for a fixed duration, achieve undetectable measurable residual disease (uMRD) in the vast majority of patients. On these grounds, a time-limited MRD-driven strategy, a previously unexplored scenario in CLL, is being attempted. On the other side of the spectrum, novel genetic and non-genetic mechanisms of resistance to targeted treatments are emerging. Here we review the response assessment criteria, the evolution and clinical application of MRD analysis and the mechanisms of resistance according to the novel treatment strategies within clinical trials. The extent to which this novel evidence will translate in the real-life management of CLL patients remains an open issue to be addressed.

## 1. Introduction

The clinical and biological heterogeneity of chronic lymphocytic leukemia (CLL) translates in a variable survival expectancy, ranging from few years up to decades. Despite this, the disease is still considered uncurable [1].

The evolution of CLL treatment strategies in the last ten years has been impressive [2]. On the one hand, continuous therapies with BTK inhibitors (BTKi) achieve a prolonged and sustained control of the disease. On the other hand, the BCL2 inhibitor venetoclax and anti-CD20 monoclonal antibodies or, more recently, ibrutinib plus venetoclax combinations, given for a fixed duration, achieve undetectable measurable residual disease (uMRD) in the vast majority of patients [3].

Finally, the third road that has been opened is a time-limited MRD-driven strategy, firstly attempted in the relapsed/refractory (R/R) setting and now in treatment-naïve (TN) patients [4].

The recent evolution in CLL therapy has led to a progressive adjustment in the way clinicians evaluate the response and the goals of treatment compared to the paradigm of the CIT era. On the other side of the spectrum, in patients failing to achieve a response or relapsing under target agents, novel mechanisms of resistance to treatment are emerging.

Here we review the evolution in response assessment criteria, the clinical application of MRD analysis, and the emerging mechanisms of resistance according to the novel treatment strategies within clinical trials and in the real-life management of CLL.

## 2. How to Assess Response to Treatment: Recommendations from the International Guidelines

Conventionally, response evaluation after treatment should include physical examination and evaluation of the peripheral blood (PB) and bone marrow (BM). Indeed, according to the most recent European guidelines [1,5], two groups of parameters need to be evaluated: group A, assessing the lymphoid tumor load (lymphadenopathies, hepatosplenomegaly, and lymphocytosis) and constitutional symptoms, and group B, assessing the hematopoietic system (i.e., hemoglobin, platelet count, and BM infiltration).

### 2.1. BM Evaluation

Within clinical trials, a unilateral BM aspirate and a biopsy are recommended before initiating treatment, in order to better define the response afterwards.

Outside clinical trials, BM biopsy is not mandatory before and after treatment, but may be helpful in certain conditions, i.e., to assess the cause of cytopenias due to treatment toxicity or disease progression [1,5].

### 2.2. Imaging

The staging of CLL does not use computed tomography (CT) scans but relies on physical examination and blood counts; thus, enlarged lymph nodes, if detected only by CT scan, do not change the Binet or Rai stage [1]. Patients with Rai stage 0 but abdominal disease detectable by CT scans may have a more aggressive course [6], but more evidence is needed to introduce CT scans for the routine initial evaluation of CLL patients.

Furthermore, the majority of relapses or progressions in CLL are detected by physical examination and blood counts, and not by imaging studies [7]; the decision for treatment of relapse is determined by imaging studies in only 1% of patients [7].

In clinical trials, where the treatment intent is to maximize the overall response rate, total-body CT scans are recommended before the start of therapy and at response assessment after treatment, if abnormal at baseline, to evaluate the response [1].

For the evaluation of efficacy of novel treatments with continuous administration within clinical trials, more than one CT scan might be necessary [5], but it should be considered that additional, repetitive CT scan monitoring is usually not clinically relevant and potentially harmful for the patient.

Nevertheless, imaging in the real-life of CLL patients’ management may be overused in some countries [8].

### 2.3. Measurable Residual Disease

Following the evidence that MRD has a strong prognostic impact following CIT [9,10], the EMA (European Medicines Agency) has accepted uMRD in CLL patients in clinical complete response (CR) (=MRD response rate) after induction therapy as a surrogate end-point of efficacy in randomized studies designed to show superiority in terms of PFS (MA, European Medicines Agency, December 2015).

MRD analysis in CLL clinical trials has been recently reviewed [11], being a secondary end-point in several clinical trials or even a primary end-point [12].

The employment of MRD as a decision tool in clinical practice is not allowed, except for patients who undergo allogeneic stem cell transplant (SCT), where a positive MRD signal may trigger the reduction of immunosuppressive therapies, the administration of donor lymphocyte infusions, or the start of antileukemic maintenance therapy [5]. Therefore, MRD monitoring after therapy is generally not recommended outside clinical studies. Moreover, not every CLL patient needs to achieve an uMRD (see below) [4].

### 2.4. Timing of Response Assessment

The timing of response assessment for therapies with a defined treatment duration should be at least 2 months after completion of therapy. In clinical trials, any response (CR or partial remission, PR) should be sustained for at least 2 months before using this response in the assessment. MRD assessment should not be limited to patients in CR, as practiced in some studies, since patients in PR may also achieve uMRD, as shown after CIT or after venetoclax-based combinations [13,14,15].

MRD in the BM may be performed at least 2 months after the patient has cleared MRD from the PB [1].

## 3. Methodological Considerations of MRD Testing

A considerable effort has been made by a consensus of experts to establish a nomenclature and give recommendations regarding methodology, assay requirements and in which tissue to assess MRD, and timing and frequency of assessment, in clinical trials vs. clinical practice [16].

Conventionally, MRD is defined as uMRD in cases with fewer than 1 CLL cells in 10,000 leukocytes (<0.01% or <10^−4^ or MRD4). MRD4 historically proved to be an independent prognostic factor after first-line CIT [3,9,10].

MRD is defined intermediate in cases with 1–99 CLL cells per 10,000 leukocytes (<1% and ≥0.01% or <10^−2^ and ≥ 10^−4^) and positive in cases with ≥100 CLL cells per 10,000 leukocytes (≥1% or ≥10^−2^).

**Figure 1:**
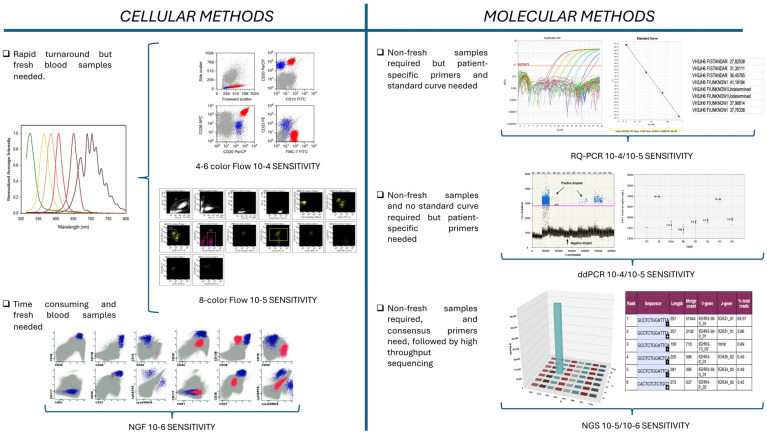
Methods for MRD monitoring in CLL. Abbreviations: NGF, next-generation flow cytometry; RQ-PCR, real-time quantitative polymerase chain reaction; ddPCR, digital droplet PCR; NGS, next-generation sequencing.

Currently, the internationally standardized methods for MRD analysis in CLL are multi-color flow cytometry (MFC) and real-time quantitative polymerase chain reaction (RQ-PCR). With the advent of improved methodologies, such as next-generation flow cytometry (NGF), next-generation sequencing (NGS), and digital droplet PCR (ddPCR), each with their specific advantages/disadvantages, the sensitivity at which MRD can be measured is continuously increasing [16] (Figure 1).

For flow cytometry, the evaluation of residual cells is mainly based on the differential expression of surface antigens between CLL and normal B cells. The four-color assay is the conventional gold standard, but six- and eight-color flows are currently used [17,18,19,20]. The number of acquired events is a crucial aspect to reach the sensitivity of at least 10-4 (MRD4): at least 500,000 events need to be acquired in each tube with the identification of at least 20 clustered events showing a CLL-related immunophenotype.

It is worth noting that, in all the FILO trials with fludarabine-cyclophosphamide-rituximab (FCR)-based CIT, a MCF MRD assay with increased sensitivity (0.7 × 10^−5^) applied to PB samples provided additional prognostic information and demonstrated the possibility of avoiding BM samples [21].

MFC is an optimal tool for rapid turnaround, but fresh blood samples are needed [19,20].

During recent years, NGF methods were proposed to reach higher sensitivity (close to 10^−6^) compared to MFC, with faster and reproducible results, with a great applicability (>95%). However, the acquisition of many cells is always needed to reach a high sensitivity, and fresh material should be analyzed within 24 h after sampling. These systems require significant software skills, limiting their application to few specialized laboratories, and few experiences have been conducted on CLL [22].

RQ-PCR MRD assessment is based on immunoglobulin rearrangements to identify a molecular target on which patient-specific sequences are designed and used for MRD monitoring after treatment [18,22,23,24,25,26].

The method reaches a detection limit (LOD) of 10^−5^ (1 leukemic cell in 100,000 normal lymphoid cells). However, for each patient-specific primer set, the LOD must be determined, as well as the conditions of amplification on a standard curve built by the diagnostic material that is serially diluted with normal mononuclear cells [18,22,23,24,25,26].

Despite its high sensitivity, the need for a standard curve for all MRD evaluations can limit the possibility of monitoring patients over time due to the availability of sufficient diagnostic material.

MFC [17,19] and RQ-PCR techniques [27,28] have undergone clinical validation and cross-laboratory standardization in Europe.

In comparison to RQ-PCR technology, the ddPCR technique is based on the principle of partitioning the sample into many nano-PCRs (droplets) containing single, few, or no target sequences. The target concentration is calculated by applying Poisson’s statistics. ddPCR can detect and quantify molecular targets with high accuracy, particularly when the disease level is very low [29,30]. ddPCR MRD analysis has been investigated in several lymphoid malignancies (i.e., acute lymphoblastic leukemia, mantle cell lymphoma, and follicular lymphoma), showing a great rate of concordance with RQ-PCR MRD assessment and proving to be a promising tool to further refine MRD monitoring [31,32,33,34]. However, no data have been so far reported in CLL [35].

Another molecular technology for MRD analysis is the NGS. This approach uses consensus primers for the amplification of immunoglobulin genes followed by high-throughput sequencing for quantification of the sequence identified in diagnostic samples [36,37,38,39,40,41]. Also, for NGS-MRD analysis, as for NGF, a LOD of 10^−6^ (1 leukemic cell in 1,000,000 normal lymphoid cells) is achievable when high amounts of DNA are used [16].

The value of this method in MRD monitoring is expanding across several hematological malignancies, including CLL [36,37,40,41,42,43,44].

The NGS technology not only has the potential to overcome some limitations of PCR-based methods but also enables the analysis of the genetic heterogeneity, which may contribute to a better understanding of the disease biology [39]. On the other hand, the availability of skilled bioinformatics is needed for analysis and interpretation.

Currently, no established guidelines for NGS and ddPCR MRD analysis have so far been defined, but a major standardization effort is underway within the EuroMRD Consortium.

The value of the new technologies needs to be conclusively documented.

Finally, both PB and BM are sensitive sources of residual disease, with a concordance rate that can be affected by the type of treatment [3,4]. Indeed, after CIT [9,10] or the venetoclax and anti-CD20 monoclonal antibodies combination [14,15,41,42,43], the first MRD analysis should be performed in PB samples and, if the results are negative, expanded to the BM compartment.

After the ibrutinib and venetoclax combination, the clearance of MRD appears less dependent from the disease compartment, as shown in the CAPTIVATE trial [45,46] as well as in the GLOW trial [44]. The high concordance in uMRD between the BM and PB—assessed in the former study by MFC (94%) and in the latter by NGS (90.9–92.9% using <10^−4^ or <10^−5^ cutoff)—has the potential to reduce the need for BM aspirates in future clinical practice.

The most sensitive technologies now available could detect residual disease hidden in other compartments (spleen, liver, or lymph nodes) that can play a role in a subsequent relapse, by evaluating other sources of nucleic acids, such as plasmatic cell-free DNA/microRNA or those derived from exosomes that in the future could be explored as biomarkers in CLL, as it is the case for solid tumors [47,48].

## 4. Evaluation of Response

Over recent years, the first-line therapeutic approach explored in CLL clinical trials has followed two main roads: continuous anti-BTK inhibitors, (BTKi)-based therapy, whose efficacy is independent from the achievement of uMRD [49,50], and fixed-duration schemas with uMRD as a relevant end-point of efficacy based on venetoclax and anti-CD20 monoclonal antibodies [41,42,43] or on venetoclax and ibrutinib, associated with progressively higher rates of MRD negativity [12,44,46].

### 4.1. Continuous Therapies

Since the introduction of BTKi with a continuous treatment approach (i.e., until disease progression or unacceptable toxicity), some changes have been introduced in response categories and in the timing of response assessment.

PR with lymphocytosis (PR-L) is a new category, due to the ibrutinib-induced lymphocytosis that occurs in 20% of patients, and is usually transient in most patients and resolves within 12–18 months [1,51,52]. This should prevent categorizing these patients as treatment failures and inducing early ibrutinib discontinuation.

Moreover, response to ibrutinib in the first line improves over time, with an increase of CR occurring beyond 12 months of treatment and up to over 30% after 5 years of treatment [53].

Therefore, according to the guidelines, the assessment of response should be performed at least 2 months after patients achieve their maximum response; in this case, it is not necessary to interrupt therapy for response assessment [1]. The assessment of “maximum response” can be not so univocal, although it is defined as a treatment phase in which no additional improvement is seen during at least 2 months of therapy.

Accordingly, within clinical trials, CT scans should be performed at the time point of clinically evaluated maximal response or, alternatively, at a time point defined by the protocol [1].

For patients on continuous BTK inhibitor monotherapy or in combination with anti-CD20 monoclonal antibody rituximab, only 10% might reach uMRD at some stage. The combination of ibrutinib + rituximab achieved uMRD in 6.2% of patients in the GIMEMA LLC1114 trial and in 27% of patients in CR [54].

Ibrutinib + rituximab achieved uMRD in 7.9%, 4.2%, and 3.7% of patients at 12, 24, and 36 months, respectively, in the ECOG E1912 randomized trial [49]. In this trial, PFS did not significantly differ by uMRD status in the ibrutinib + rituximab arm at variance from the CIT arm with FCR [49].

Therefore, for patients on indefinite ibrutinib-based therapy, MRD monitoring is unnecessary and is not a prerequisite for a prolonged PFS. Continuation of single-agent ibrutinib is required to maintain treatment efficacy.

It is possible to achieve higher rates of uMRD with BTKi only combining them with other agents, such as obinutuzumab. In first-line treatment, ibrutinib + obinutuzumab led to uMRD in the PB in 33% of patients, higher than chlorambucil + obinutuzumab [55]. Acalabrutinib + obinutuzumab (A+O) (ELEVATE TN) led to a higher proportion of uMRD than acalabrutinib only (uMRD in 38% vs. 10% of patients with CR/CRi, respectively) [50].

Criteria to define BTKi failure and when to switch to another treatment are not well defined in the current guidelines. Within clinical trials, in patients who lose response during continuous BTKis, it is recommended to stop treatment. iwCLL guidelines do not recommend starting a new line of therapy at relapse, but instead applying the criteria of disease progression even for further lines of treatments [1].

### 4.2. Fixed-Duration Therapies

Venetoclax-based fixed-duration therapies result in uMRD rates in a high percentage of patients.

The first evidence is derived from the combination of venetoclax with rituximab (Ven+R) administered for 2 years in R/R patients in the Murano trial [14,15,56] and from the combination of venetoclax with obinutuzumab (Ven+O) given for 1 year in the front-line setting in the German CLL14 trial [41,42,43].

In both trials, MRD was assessed by RQ-PCR with the following quantitative cutoff: uMRD, <10^−4^; low MRD, 10^−2^ to 10^−4^; high MRD, >10^−2^.

In the Murano trial, the achievement of an uMRD in PB at the end of Ven+R combination treatment (62.4%) predicted longer PFS [15], and an uMRD in PB at the end of a 2-year therapy (end of treatment, EOT) in patients with no progressive disease (PD) (64%) predicted longer PFS and OS [56]. Patients with uMRD at EOT had superior OS vs. those with high MRD+. Median time from uMRD to MRD positive conversion was 19.4 months, and the median time from MRD conversion to PD, reported in 40% of patients, was 25.2 months [56].

In the CLL14 trial, at the EOT (month +15, M15), uMRD (<10^−4^) rates by RQ-PCR were 76% in the PB and 57% in the BM [43]. The achievement of uMRD was an early event, occurring by cycle 7, and was sustained over time. uMRD4 by RQ-PCR significantly predicted PFS.

In the same trial, MRD was also evaluated by NGS [41,42]. At the EOT, MRD assessed by NGS significantly predicted PFS and OS. No differences in PFS were detected between uMRD6, uMRD5, or uMRD4 by NGS [42]. This was not the case in a retrospective study after FCR, where uMRD < 10^−6^ by NGS was suggested to improve the outcome prediction vs. the conventional flow cytometry uMRD4 [40].

In the CLL14 trial, 18 months after the EOT, the PB uMRD4 rate was 47% for VenG and decreased thereafter, with 36.1% PB uMRD4 at 48 m after EOT. The conversion rate from uMRD4 (<10^−4^) to detectable MRD (>10^−4^) was 21 months [41].

In the CLL14 trial, high rates of uMRD at EOT were documented even in patients with high-risk features. Indeed, PB uMRD4 rates at EOT were 79% for unmutated IGHV, 74% for mutated IGHV, and 68% for TP53 deleted/mutated cases, suggesting a similar depth of remission in all subgroups after Ven+O treatment.

However, despite similar rates of uMRD at EOT, the growth dynamics of clones in patients with high-risk disease features were faster than in other patients. Therefore, EOT uMRD status alone might yield limited information on the durability of deep remissions in these high-risk subgroups [41].

These observations support the concept that MRD kinetics appear more informative than a punctual evaluation and can differ according to disease biology.

Along this line, after FCR, patients with BM uMRD at EOT but unmutated IGHV showed a shorter time to conversion to MRD+ than patients with mutated IGHV [57].

The presence of genetic complexity was associated to a more rapid regrowth of the CLL clone in the Murano trial, and this translated into a shorter PFS [15].

On the other hand, patients with mutated IGHV have a good outcome with any regimen; thus, especially for these patients, other clinical end-points, such as quality of life, should be taken into consideration [4].

## 5. Optimizing the Use of MRD Testing: From Fixed-Duration to Time-Limited Therapy

The best outcome for a CLL patient needing therapy is obtaining a CR with uMRD. Venetoclax, alone or combined with anti-CD20 antibodies, demonstrated a high rate of uMRD, representing the first non-chemotherapy-based, fixed-duration regimens in CLL [43,56,58]. Since both venetoclax and BTK inhibitors target key CLL vulnerabilities, combining these agents appears an obvious strategy to explore new ways to achieve higher and durable uMRD CRs. These agents show a differential “compartment effect” as BTKi mobilize cells from protective niches in lymph nodes [59], while venetoclax hits cells in blood and BM [60]. Preclinical studies investigating BCL2 and BTK dual targeting showed synergistic effects [60], suggesting that this strategy can identify clinically useful combinations. Initial phase II trials demonstrated MRD responses of approximately 75% in first-line therapy and 58% in relapsed/refractory CLL after ibrutinib plus venetoclax [61,62]. Results from these fixed-duration trials have opened the way to MRD-guided strategies with potential for effective time-limited therapy. This is crucial because it can allow for a shorter therapy duration, side effects reduction, and cost effectiveness. MRD-guided studies are ongoing to establish when to stop treatment as well as when to resume it again, in case of detectable MRD or progression. To date, the use of MRD as treatment guide in routine practice is not recommended, and dedicated randomized studies should demonstrate the long-term benefits of MRD-guided treatment interruption, continuation, or intensification.

### 5.1. Fixed-Duration Treatments to Maximize MRD Responses

The phase 3 GLOW study provided the first prospective data on MRD outcomes in older or unfit patients treated with ibrutinib plus venetoclax versus obinutuzumab-chlorambucil as a first-line treatment [63]. This study demonstrated improved depth of response and sustained uMRD during the first-year post-treatment, threefold higher for ibrutinib-venetoclax (84.5%) than obinutuzumab-chlorambucil (29.3%) in PB. In patients achieving uMRD 3 months after the end of ibrutinib and venetoclax, the PFS rate was 82% compared to 73% in patients with detectable MRD (dMRD) at 3 years post EOT. The uMRD rates in the GLOW trial were consistent with those reported in the non-randomized CAPTIVATE trial that tested similar fixed-duration treatment in younger and fitter patients [45,46,64]. A different trial assessing venetoclax and ibrutinib for 24 cycles revealed that a 2-log reduction at the end of cycle 3 or an MRD of less than 1% in BM were associated to a higher likelihood of achieving uMRD at the end of cycle 12 [65].

In a follow-up analysis, the GLOW study showed a strong interaction between MRD outcome and IGHV repertoire [66]. Contrary to expectation, uMRD rates were higher and were achieved earlier in patients with unmutated versus mutated IGHV CLL (59.7% vs. 40.6%), although this incidence did not translate into a greater PFS benefit. One possible explanation is the increased sensitivity of unmutated IGHV cells to ibrutinib, which depends on their enhanced B-cell receptor (BCR) signaling and heightened BTK-dependent proliferation. Two years after EOT, the PFS rate was more than 90% for patients with mutated IGHV, whether they had detectable or undetectable MRD at that time, and for patients with unmutated IGHV and uMRD. By contrast, only 67% PFS was reached in dMRD and unmutated IGHV patients at the same time point. Thus, the use of BCL2 plus BTK inhibitors may mitigate the predictive value of post-treatment MRD in favor of IGHV assessment, since it dictates the regrowth and response kinetics of MRD. This is consistent with earlier data on the venetoclax and obinutuzumab combination, which showed a correlation between MRD doubling time and the presence of high-risk genetics such as IGHV or complex karyotype [41].

The value of MRD testing in fixed-duration therapy has been further investigated in the phase 3 GAIA-CLL13 trial comparing venetoclax-based combinations to conventional CIT in fit patients without *TP53* aberrations [12]. The co-primary end-point analysis of the trial showed superior uMRD rates for venetoclax-obinutuzumab (GV) and GV + ibrutinib (GIV) compared to venetoclax-rituximab (RV) and CIT. The percentages of uMRD in GV and GIV arms were among the highest reported in first-line therapy (86.5% and 92.2%, respectively), being slightly higher than fixed-duration venetoclax plus ibrutinib in other trials [46,61]. Attainment of uMRD was associated with longer PFS among patients reaching a CR or even a PR. Exploratory MRD analyzed by NGS [67] revealed 22.7% (CIT), 23.6% (RV), 60.3% (GV), and 66.2% (GIV) uMRD < 10^−6^ in PB at month 15. NGS-based MRD appeared to improve prognostication in patients with uMRD < 10^−4^ by conventional MFC, further corroborating the prognostic value of uMRD in fixed-duration treatment [9]. Whether doublet fixed-duration therapy is noninferior to a continuous BTK inhibitor is currently being addressed in the prospective phase 3 CLL17 trial.

In addition to the GAIA trial, other studies are looking into whether a triple regimen consisting of venetoclax, a BTKi, and an anti-CD20 antibody is even more advantageous than doublets or continuous BTKi in terms of response depth and durability. In a study from the Ohio State University, a combination regimen of obinutuzumab, ibrutinib, and venetoclax for a total of 14 cycles resulted in BM uMRD in 67% of untreated CLL [68], which was unexpectedly lower than the GAIA trial and even venetoclax-antibody doublet therapies [43]. Similar triple combinations were tested in first-line CLL with *TP53* aberrations in the CLL2-GIVe trial, which found 81% uMRD rates in PB [69]. Similar uMRD rates were found after one-year treatment in the phase 2 AVO study [70], using the acalabrutinib, venetoclax, and obinutuzumab combination in previously untreated CLL, and the BOVen study [71], which used zanubrutinib, venetoclax, and obinutuzumab. At this stage, the value of triplets remains unclear as none of the trials definitively show that a triple combination significantly raises uMRD rates over doublets. Yet, a number of patients still have detectable MRD levels after treatment completion. Most importantly, some of the benefits of the addition of CD20 antibodies and BTKis to venetoclax are neutralized by the need for dose reductions and discontinuation for adverse events.

### 5.2. Time-Limited MRD-Guided Treatment Strategies

More recent trials have focused on unexplored strategies of treatment intensification, de-escalation, or discontinuation according to MRD status.

This approach was firstly tested in the phase 2 HOVON141/VISION trial [72] that investigated MRD-guided treatment cessation after a fixed duration of ibrutinib and venetoclax in R/R CLL. Specifically, uMRD patients were assigned to either continue ibrutinib or stop therapy to demonstrate similar PFS at month 27, reinforcing the idea of time-limited therapy guided by MRD.

The phase 2 CLARITY trial enrolled patients previously treated with at least one line not including BCL2 or BTK inhibitors to receive venetoclax plus ibrutinib [62]. The study included MRD-driven stopping rules so that patients requiring 6 months to reach uMRD in BM discontinued treatment after 12 overall cycles, whereas patients who, for instance, required 12 months to reach uMRD were treated for 24 months in total. Two-thirds of patients had uMRD after 6 months of treatment and received treatment for a total of 12 months, whereas 58% of patients reached uMRD after 12 months. After 3 years, responses were sustained even though many patients had discontinued treatment due to uMRD achievement.

In the same R/R settings, venetoclax therapy driven by MRD was explored in the phase 2 IMPROVE trial [73]. After receiving venetoclax monotherapy for 12 cycles, patients were instructed to either stop treatment if uMRD was reached at cycle 12, or to continue receiving venetoclax in combination with ibrutinib if MRD was detectable. A combination was administered until cycle 24, uMRD4, progression, or unacceptable toxicity. After 1 year of venetoclax, 45% of all patients had uMRD and discontinued treatment, whereas 50% of the patients continued with venetoclax plus ibrutinib. The uMRD rate at month 24 was 84%, indicating that BTKi intensification aids in MRD clearance. The most recent follow-up showed 53% of patients with MRD relapse with no clinical disease. Serial measurements of MRD dynamics during follow-up may improve the prognostic utility of individual MRD snapshots at particular time points.

The logical extension of this research in the R/R setting was to see whether ibrutinib and venetoclax combination could be used in first-line treatment. The prognostic impact of uMRD at the EOT has become evident from the randomized part of the CAPTIVATE study in which MRD testing was used to assign TN patients to receive additional treatments after venetoclax plus ibrutinib [45]. Patients with uMRD were further randomized to continue ibrutinib versus placebo, demonstrating that uMRD rates were sustained 3 years post-randomization in both groups and suggesting that patients do not benefit from maintenance with ibrutinib after reaching uMRD [74]. Conversely, in dMRD patients after a 12-cycle combination, increases in uMRD were greater with continued ibrutinib plus venetoclax versus ibrutinib alone. Nevertheless, estimated 30-month PFS rates were similar across groups, suggesting that dMRD patients do not benefit from intensive maintenance and that ibrutinib monotherapy could be enough to prevent relapse [45].

Serial monitoring of MRD has been used to guide therapy in the ibrutinib plus venetoclax arm of the phase 3 FLAIR study, which compared FCR, ibrutinib, ibrutinib plus rituximab, and ibrutinib plus venetoclax in TN CLL [75]. This study showed that the rate of uMRD significantly increased after an additional year of combination therapy, and 90.6% patients achieved uMRD in the PB at up to 5 years treatment intensification, demonstrating that extended treatment with BCL2 plus BTK inhibitors can deepen remissions. Using MRD to direct the duration of this combination maximizes outcomes with 97.2% 3-years PFS, demonstrating unprecedented efficacy results, superior to previous phase 3 trials. Longer follow-up is still required to understand whether patients derive any benefit from longer combination therapy and to turn ibrutinib plus venetoclax with MRD-guided therapy as a new gold standard for CLL treatment.

Determining the optimal doublet combination partners and duration remains a key question for frontline CLL therapy. The ongoing MAJIC clinical study [76] will presumably answer this issue by comparing, in a randomized, prospective way, whether MRD-driven finite therapy with acalabrutinib plus venetoclax achieves noninferior efficacy to MRD-driven finite therapy with venetoclax plus obinutuzumab based on a primary end-point of PFS.

## 6. Mechanisms of Resistance to Target Therapy in CLL

**Figure 2:**
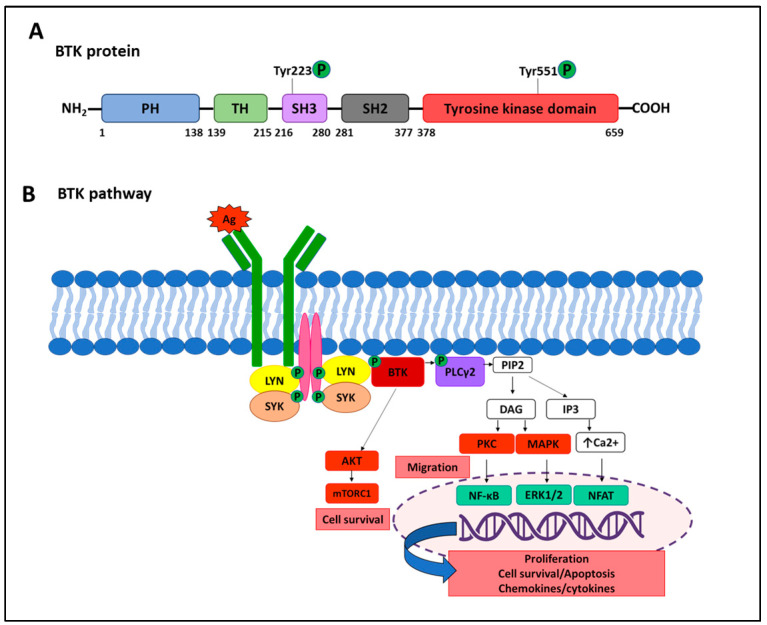
BTK structure and pathway activation. (**A**) Schematic representation of BTK protein consisting of 659 amino acids organized in five domains: the pleckstrin homology (PH) domain, the proline-rich TEC homology (TH) domain, the SRC homology (SH) domains named SH3 and SH2, and the tyrosine kinase domain. The PH domain has a binding affinity for phosphatidyl inositol trisphosphate (PIP3) generated by the PI3K pathway activation. Consequent to PIP3 engagement, BTK can translocate to the cell membrane. The TH domain is important for optimal activity and stability of the protein through Zn^2+^ ion binding. The SH domains are involved in protein–protein interactions [77]. (**B**) BTK pathway activation. Upon antigen (Ag)-mediated BCR stimulation, BTK is primed for activation by LYN (Lck/Yes novel kinase) and SYK (spleen tyrosine kinase) protein kinases, through phosphorylation of Tyr551 residue in the kinase domain. Autophosphorylation on Tyr223 in the SH3 domain allows full BTK activation. Activated BTK drives phosphorylation of phospholipase Cγ2 (PLCγ2), which catalyzes the breakdown of the membrane lipid PIP2 in the second messengers IP3 and DAG, which in turn results in calcium mobilization from endoplasmic reticulum and protein kinase C (PKC) activation, respectively. The consequences of this signal transduction are the activation of MAP kinases and transcription factors, including NF-κB, NFAT, and ERK1/2, regulating the expression of downstream genes controlling proliferation, cell survival and apoptosis, and chemokines and cytokines production. In addition, BTK can also activate the AKT/mTORC1 pathway [78,79]. BTK is involved in transducing signals from toll-like receptors (TLRs) and the chemokine receptors through NF- κB [80,81], leading to anti-apoptotic and proliferative signals. Moreover, BTK is critical for BCR- and chemokine-controlled, integrin-mediated retention and/or homing of CLL B cells in the supporting lymph node and bone marrow microenvironments [82]. Note that not all pathways upstream and downstream of BTK are shown.

**Figure 3:**
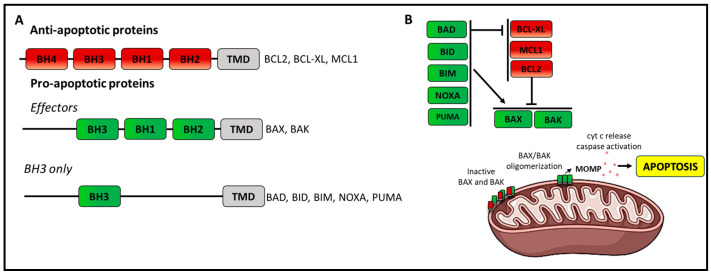
BCL-2 protein family. The BCL2-family proteins are key regulators of mitochondrial apoptosis with anti-apoptotic (red) and pro-apoptotic functions (green). (**A**) The anti-apoptotic members (i.e., BCL2, BCL-XL, MCL-1, etc.) are characterized by the presence of four BH domains and a transmembrane domain (TMD), which anchor them to organelle membranes. The pro-apoptotic BCL2 family members can be further divided into two subgroups: the multi-BH-domain effector proteins which contain three BH domains (i.e., BAX and BAK) and the BH3-only initiator proteins (BAD, BID, BIM, NOXA, PUMA, etc.) containing only the BH3 domain. (**B**) The balance and interactions between members of the BCL2 family determine whether apoptosis may occur. When pro-apoptotic proteins are sequestered by binding to anti-apoptotic members, the balance shifts towards cell survival [83]. Apoptosis initiates when stress signals upregulate BH3-only proteins, which can directly activate pro-apoptotic effectors or engage anti-apoptotic members freeing pro-apoptotic BAX and BAK [84]. When the number of free pro-apoptotic proteins reaches a certain level, oligomerization of pro-apoptotic BAX and BAK is triggered with consequent permeabilization of the outer mitochondrial membrane, release of cytochrome c (cyt c), and caspase activation leading to apoptosis.

Despite remarkable clinical efficacy, several mechanisms of resistance to CLL target therapy have been identified. In the following paragraphs, we will describe the main mechanisms of resistance to BTK (Figure 2) and BCL2 pathways inhibition (Figure 3).

### 6.1. Resistance to BTK Inhibitors

Current FDA-approved BTKi for CLL treatment includes the first-generation inhibitor ibrutinib and the second-generation acalabrutinib and zanubrutinib. These covalent BTKis work by irreversible binding to the cysteine 481 (C481) residue within the ATP-binding site of BTK [85]. Noncovalent BTKi not requiring the C481 site to reversibly bind to BTK has been also developed to improve the pharmacologic properties of covalent BTKi.

#### 6.1.1. Target Modification Mediating Resistance to Ibrutinib

**Figure 4:**
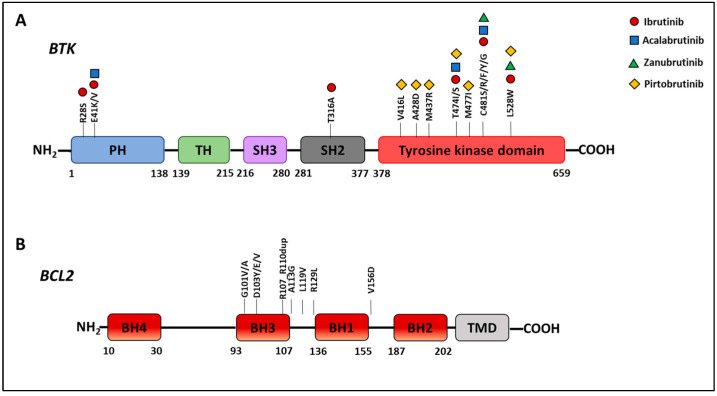
Resistance-associated mutation of the target in (**A**) *BTK* and (**B**) *BCL2* genes. PH, Pleckstrin homology domain; TH, TEC homology domain; SH2/3, Src homology domain; BH1-4, BCL-2 homology domain 1-4; TMD, transmembrane domain.

The first BTKi resistance mechanism was described in ibrutinib-treated patients in which point mutations affecting BTK (Figure 4; Table 1) and PLCγ2 were identified [86,87,88,89,90,91].

Whole-exome sequencing (WES) at baseline and at relapse of ibrutinib-resistant patients revealed mutations affecting the C481 residue of BTK. The most frequent alteration was a serine substitution (C481S), which maintains the kinase activity of BTK but disrupts the binding capability of ibrutinib [86,87]. Substitutions of the C481 position to amino acids such as tyrosine, arginine, phenylalanine, and glycine have also been described with lower frequencies [91,92,93,94].

Mutations of BTK have also been reported at non-C481 sites. Amino acid substitutions to isoleucine or serine have been described for the gatekeeper residue T474, crucial for inhibitor binding, and they co-occur with C481S mutations [93,95,96]. R28S and E41K mutations, interfering with membrane localization of BTK, have also been identified [97,98]. A structurally novel T316A mutation, which does not directly interfere with ibrutinib binding to BTK but confers resistance to ibrutinib similarly to the C481S mutation, has been described [90,99].

Multiple subclones carrying different mutations arose independently, leading to subclonal heterogeneity of resistant disease [91]. The heterogeneous landscape of BTK mutations in CLL progressing on BTKi has important clinical implications as many sites determine many resistant clones.

Active BTK directly phosphorylates PLCγ2, triggering its activation and less frequent gain of functional mutations of PLCG2 [96,100], which elicit BTK-independent activation of PLCγ2 after BCR engagement, implying a BTK-bypass pathway that has been described in resistant patients. Activation of mutant PLCγ2 was indeed demonstrated to be functionally dependent on LYN and SYK kinases [100]. Interestingly, PLCG2 mutations often co-exist with the BTK mutations [86,91,99,100,101].

#### 6.1.2. Target Modification Mediating Resistance to Acalabrutinib and Zanubrutinib

Mutations of the target have been implicated in resistance also for the newer covalent BTKi acalabrutinib and zanubrutinib (Figure 4; Table 1).

In the first study reporting CLL relapse on acalabrutinib, resistance was shown to be mediated predominantly by BTK mutations with 69% of cases carrying a C481S/R/Y mutation and one out of sixteen having both C481S and T474I mutations. In the same cohort, *PLCG2* mutations have also been detected [102]. In the head-to-head trial of acalabrutinib versus ibrutinib, common mutations were observed with both treatments, but patterns of mutation frequency, variant allele frequency (VAF), and uncommon *BTK* variants were different [103]. The rate of BTK mutations was higher in acalabrutinib- (66%) versus ibrutinib-treated patients (37%) while *PLCG2* mutations occurred with higher rate in the ibrutinib cohort (20% vs. 6%). Interestingly, a new E41V mutation within the PH domain of BTK was seen in one patient in the acalabrutinib cohort [103].

A L528W mutation of BTK was reported in four patients progressing on zanubrutinib. These patients had also C481 mutations at lower VAF than the L528W. The enzymatic activity of BTK L528W was significantly lower than C481S and wild-type BTK [104]. The enrichment of L528W mutations was more frequent (54% vs. 4%) in patients treated with zanubrutinib compared to ibrutinib [105]. The L528W mutation reduces BTK stability and decreases binding affinity, leading to resistance [106].

#### 6.1.3. Resistance to Next-Generation Noncovalent BTK Inhibitors

Noncovalent BTKis reversibly bind to BTK not requiring C481. Several noncovalent BTKis have been studied preclinically and in clinical trials including pirtobrutinib and nemtabrutinib. NGS analysis of patients under pirtobrutinib revealed BTK-resistance mutations (V416L, A428D, M437R, T474I, or L528W) outside the C481 position, conferring resistance to both noncovalent and some covalent BTKis. Interestingly, cell-based assays linked the presence of these mutation to a not-suppressed AKT activation [107]. Pirtobrutinib resistance has been associated to a T474I mutation in co-occurrence with a new M477I inactivating mutation acquired at progression [108].

The kinase-dead L528W, M437R, and V416L mutations, though impeding drug binding and disabling enzymatic activity of BTK, still enable BCR signaling, suggesting the involvement of non-enzymatic activity [109]. Phosphoproteomics, kinobead assays, and BTK immunoprecipitation mass spectrometry studies revealed a physical interaction of BTK L528W with LYN and HCK for kinase activation and substrate phosphorylation [109]. This discovery encourages targeting of BTK by elimination rather than inhibition, supporting the clinical efforts on BTK degraders to overcome resistance.

#### 6.1.4. Gatekeeper Mutations and Super-Resistant Variants

The role of combined gatekeeper/C481 BTK variants in the resistance to reversible and irreversible BTKis has been shown in T474I/C481S, T474M/C481S, and T474M/C481T mutants, which resulted in insensitivity to irreversible BTKis while variable resistance patterns have been revealed for reversible inhibitors [95]. This suggests that newer noncovalent BTKi might overcome the resistance observed with covalent BTKi even in the context of super-resistant mutants, pointing to the possibility to re-treat a resistant patient with another BTKi after relapse. In the phase 1/2 BRUIN study, pirtobrutinib allowed the clearance of C481 mutations of ibrutinib resistance while new BTK mutations such as L528W, V416L, A428D, D539G, and Y545N and gatekeeper mutations like T474I/F/L/Y were detected at pirtobrutinib resistance [110]. Further studies are necessary to determine which noncovalent BTKi resistance mutations are able to circumvent or not, as well as to determine their efficacy across a broader population in first-line settings to guide therapy choice and sequencing.

#### 6.1.5. Alternative Genetic Mechanisms of Resistance to BTKi

The evidence that *BTK* and *PLCG2* mutations were absent in one third of relapsed patients suggests additional resistance mechanisms [111]. *TP53*, *ATM*, *EGR2*, *SF3B1*, *NOTCH1*, and *BIRC3* are the most commonly mutated genes at progression and relapse. These aberrations and related mechanisms, such as *EGR2* and NF-κB pathway mutations, might also cooperate with *BTK/PLCG2* mutations in determining progression on ibrutinib. In *BTK* unmutated cases, the more frequently detected mutations affected *BIRC3* and *NFKBIE*, suggesting that aberrations of the NF-κB pathway can lead to progression and drug escape [111].

Apart from gene mutations, genetic resistance to ibrutinib in CLL has also been associated with chromosomal aberrations such as gain in the 2p and 8p deletions [112,113]. The gain in the 2p region has been related to in vitro drug resistance in CLL including ibrutinib, as several genes in this region are important in B-cell drug resistance. These include the proto-oncogene *MYCN*, the NF-KB subunit *REL*, the nuclear transporter exportin-1 *(XPO1)*, and the proto-oncogene *BCL11A* [112]. The 8p deletion causes loss of a gene encoding the TRAIL receptor, resulting in insensitivity to TRAIL-mediated cell death in ibrutinib-resistant CLL [113].

#### 6.1.6. Non-Genetic Mechanisms of Resistance to BTKi

Various observations, including the timing of mutation appearance, subclonal heterogeneity, and the presence of residual clonal lymphocytosis in ibrutinib-responding patients, suggest that resistance can be due to non-genetic mechanisms. These include epigenetic mechanisms, bypass pathway activation, and a supportive role of tumor microenvironment (TME).

Shaffer et al. recently proposed a mechanism through which tumor cells rewire the BCR-driven NF-κB signaling. Authors demonstrated that resistant cells showed increased interaction of PLCγ2 with RAC2 in place of BTK to sustain NF-κB activity [114].

The ibrutinib clinical efficacy was recently associated with reduced NOTCH1 activity that deepened over time. Conversely, NOTCH1 signaling was restored at relapse and remained activated in cells from ibrutinib-resistant CLL. Thus, NOTCH1 activation represents an alternative mechanism underlying acquired ibrutinib resistance, independent of mutations [115].

Also, signals from TME have been related to BTKi resistance. Ibrutinib treatment was associated with the reduction of different cytokines, excluding IL-4 and IL-6, that preserve their capacity to upregulate MCL-1 and BCL-xL for cell survival [116,117].

High surface expression of CD49d and CD79b correlated with resistance to acalabrutinib [118]. CD49d may contribute to resistance by enhancing adhesion of cells to TME and may serve as a predictive marker.

Whole genome methylation sequencing in the ACE-CL-001 trial (NCT02029443) revealed reduced IL-15 methylation correlating with acalabrutinib resistance in CLL. Similar to BTK, IL-15 signaling targets the NF-kB pathway, indicating that alternative ways of NF-kB activation may affect response to BTKi [119].

### 6.2. Resistance to Venetoclax

Anti-apoptotic proteins like BCL2 are often overexpressed in cancer cells and maintain survival by sequestering pro-apoptotic proteins (Figure 3). BCL2 overexpression is a hallmark of CLL pathogenesis that depends on different mechanisms, including miR-15 and miR-16 loss, hypomethylation of the BCL2 gene, and upregulation of STAT3 [120,121,122,123]. Venetoclax is the first-in-class BH-3 mimetic with high affinity and selectivity for BCL2 [124,125]. Venetoclax acts by competing for binding to BCL2 with BH3 pro-apoptotic proteins. Consequent to interaction with BCL2, enough BIM and BAX are available to induce apoptosis. Multiple mechanisms have been proposed to explain resistance to venetoclax, including target mutations (Figure 4), upregulation of anti-apoptotic BCL2 family members, BAX mutations, and microenvironmental signals [126].

#### 6.2.1. Target Modification Mediating Resistance to Venetoclax

The first point mutation identified in venetoclax-resistant patients was a Gly101Val mutation of the BCL2 gene. This mutation decreases the affinity of venetoclax for BCL2 by 180-fold and its ability to compete with the endogenous BH3 ligands with apoptosis reduction [127]. This mutation was present in patients under venetoclax for more than 2 years and was undetectable in patients before starting any therapy. In a trial of venetoclax-treated CLL carrying 17p deletion, Gly101Val was found to be co-mutated with an Asp103Tyr alteration [128] occurring prior to the acquisition of the Gly101Val mutation. The Asp103Tyr alteration results in the extension of a bulky amino acid into the BCL2-binding pocket for venetoclax, resulting in binding inhibition. Additional point mutations such as Gly101Ala, Ala113Gly, Leu119Val, Arg129Leu, and Val156Asp and an insertion Arg107_Arg110dup affecting the binding groove of BCL2 [129,130,131] have also been identified. Blombery et al. demonstrated that multiple mutations in BCL2 can occur in different CLL cells in a single patient [131]. The occurrence of BCL2 mutations anticipated clinical disease progression by months [127], indicating that monitoring could be advantageous, although no data on the utility of this approach are available, and their detection should not allow practitioners to discontinue therapy, per se.

#### 6.2.2. Mutations of Pro-Apoptotic BCL2 Family Members

WES and targeted sequencing of venetoclax-resistant CLL revealed the presence of mutations affecting pro-apoptotic BCL2 family members (BAD, BAX, and NOXA) [132,133]. These mutations impair mitochondrial outer membrane permeabilization leading to apoptosis. Interestingly, approximately 30% of patients exposed to long-term venetoclax harbor BAX mutations in the non-CLL compartment together with classical mutations defining clonal hematopoiesis (CH) [134]. BAX-mutated CH uncovers a phenomenon of simultaneous hematopoietic adaptation to venetoclax therapy in both the on-target and off-target hematopoietic compartments [135].

#### 6.2.3. Mutations in Other Cancer-Related Genes

WES of CLL at the time of venetoclax resistance revealed mutations in other cancer-related genes, including *BRAF*, *BTG1*, *CDKN2A/B*, *CD274*, *NOTCH1*, *RB1*, *SF3B1*, and *TP53* [136,137]. No genetic alterations in *BCL2* or *BAX* and *BAK* were found in the same samples. BTG1 has been shown to counteract cell proliferation and to be regulated downstream of BCL2 and CDKN2A/B to increase selective pressure under venetoclax treatment. Indeed, functional analysis revealed that the co-occurrence of *BRAF* and *CDKN2A*/*B* alterations lead to a strong reduction of venetoclax sensitivity in vitro [138].

#### 6.2.4. Non-Genetic Aberrations of BCL2-Family Proteins

Venetoclax resistance is only partially explained by genetic mechanisms, as *BCL2* mutations are observed only in half of CLL on long-term venetoclax and are often found at low VAF. A co-dependence on other anti-apoptotic proteins, such as BCL-XL and MCL1, can reduce sensitivity to venetoclax, leading to resistance. Indeed, venetoclax displays a high affinity for BCL2 but not for BCL-XL or MCL-1. Increased BCL-XL and MCL-1 can capture pro-apoptotic BIM displaced from BCL2 constrains by venetoclax, thus acting as resistance factors [139]. A possible mechanism for high MCL-1 expression is the 1q23 amplification encompassing the MCL1 gene [140]. Alternatively, MCL-1 upregulation has been correlated with NF-κB activation that binds to the MCL1 promoter for transcription [141]. MCL1 can also interact with pro-apoptotic proteins like BAX or BAK, blocking their function. This interaction is counteracted by pro-apoptotic BH3-only proteins like PUMA. On these bases, it is likely that loss of BH3-only proteins might cause resistance. Indeed, functional genomic screens revealed that venetoclax resistance can be mediated by de novo methylation and silencing of PUMA [142]. Epigenetic changes of PUMA lead to increased MCL1 dependence under venetoclax exposure and disappear at stop of treatment. This implies an advantage of time-limited duration of venetoclax therapy or a drug holiday strategy that may restore drug sensitivity for venetoclax re-treatment.

#### 6.2.5. Role of Microenvironment and Bypass Pathways in Resistance

Extrinsic factors from TME can drive resistance to venetoclax as microenvironment stimuli favor the upregulation of anti-apoptotic BCL2-family proteins not targeted by venetoclax. CD40 engagement on CLL cells generates ex vivo resistance to venetoclax, activating NF-κB signaling [143]. Wnt5a from the leukemia microenvironment induced ROR1 to upregulate BCL-XL in patients who failed to clear MRD after venetoclax [144]. Furthermore, TME supports the expression of NOTCH2 and MCL-1, especially in lymph nodes, in line with data suggesting an involvement of lymph nodal niches in resistance to pro-apoptotic treatments [145]. Recently, a reduced response to venetoclax was demonstrated in CLL with trisomy 12. These patients have low levels of interferon regulatory factor 4 that led to high levels of NOTCH2 and upregulation of MCL-1. NOTCH2 silencing or treatment with the MCL-1 inhibitor AMG-176 was able to restore venetoclax sensitivity [146]. Chong et al. recently found increased levels of phosphorylated MCL-1, BCL2, and BAD in CLL at progression on venetoclax. Resistant cells displayed a reduced BCL2 dependency that was restored by treatment with the protein phosphatase 2A activator in vitro [147].

## 7. Future Directions: Conclusions

With the recent advent of more effective and chemo-free options, we are facing a rapidly changing approach in the treatment of CLL patients. In the opening era of time-limited and MRD-driven therapies, the MRD-based monitoring of response from the clinical trials will enter soon real-life daily practice.

However, in this scenario there are still some open issues.

Although uMRD rates are higher in the venetoclax-obinutuzumab-ibrutinib group of the GAIA-CLL13 trial [12], the definitive meliorative impact on outcome of the triple combination requires a longer follow-up. Other triplet combinations with acalabrutinib and zanubrutinib are being also explored in phase II trials.

Resistance mutations of the drug target are associated with disease progression on BTKi or venetoclax, and it is well established that they can be detected at low VAF many months before clinical progression. Thus, monitoring mutation occurrence may be clinically relevant both in terms of uMRD and treatment sequencing. Indeed, while the presence of these mutations does not require an immediate change of therapeutic choice, it highlights patients that could benefit from closer observation. Real-life monitoring of these mutations may provide predictive biomarkers for clinicians to facilitate early therapeutic intervention to prevent relapse. Moreover, understanding the resistance may guide us toward the most promising treatments after resistance in a patient-tailored manner, providing more effective management of CLL. 

Finally, and more importantly, should all CLL patients be treated upfront with potentially eradicating treatment schedules? Considering the median age of CLL patients at treatment requirement, the impact of comorbidities, and the toxicity associated to regimens that combine BTK inhibitors and anti-BCL2 drugs, this issue is of daily relevance in clinical practice [4].

In the real-life management of CLL patients, some final considerations deserve the attention of clinicians:

- In CLL, the achievement of a CR or even of an uMRD is not always a goal or at least not a goal for everyone, with the availability of continuous treatment with BTK inhibitors;
- The radiologic assessment of response should be limited, especially in continuous treatment approaches;
- In continuous treatment regimens, the timing to assess the best response can be difficult to identify, and clinicians should keep in mind that the achievement of a CR can increase over time;
- For therapies with fixed duration, the duration of treatment is fixed and not dependent on MRD results;
- Even patients with uMRD are not cured and can relapse over time;
- With the introduction of double or triple combinations, the sequencing of therapies or re-treatment is an emerging issue, at least until one of these regimens proves to be curative;
- MRD monitoring outside clinical trials will be soon a reality, but there is a need to select the patients that require it;
- Laboratory standardization and costs still represent an issue;
- Re-treatment remains guided not by MRD conversion but by clinical criteria for disease progression.

MRD may guide treatment duration and intensity in the near future, and data demonstrating that such modification leads to improved outcomes are upcoming.

## Figures and Tables

**Table 1:** Resistance-associated mutations to *BTK* inhibitors.

Acquired BTK Mutations										
	C481S/R/F/Y/G	L528W	T474I/S	M477I	M437R	A428D	V416L	T316A	E41K/V	R28S
**Ibrutinib**	selected	selected	selected	not selected	not selected	not selected	not selected	selected	selected	selected
**Acalabrutinib**	selected	not selected	selected	not selected	not selected	not selected	not selected	not selected	selected	not selected
**Zanubrutinib**	selected	selected	not selected	not selected	not selected	not selected	not selected	not selected	not selected	not selected
**Pirtobrutinib**	not selected	selected	selected	selected	selected	selected	selected	not selected	not selected	not selected
